# Non-porous Iron Titanate Thin Films Doped with Nitrogen: Optical, Structural, and Photocatalytic Properties

**DOI:** 10.1186/s11671-017-2027-7

**Published:** 2017-04-04

**Authors:** O. Linnik, N. Chorna, N. Smirnova

**Affiliations:** Chuiko Institute of Surface Chemistry, Ukrainian National Academy of Sciences, 17 General Naumov, Kyiv, 03680 Ukraine

**Keywords:** Nitrogen-doped iron titanate films, Pseudobrookite, Landauite, Ecological photocatalysis, Optical and structural properties, XPS, 82.33Ln, 82.50.Hp, 82.45 Mp

## Abstract

The synthesized undoped and nitrogen doped iron titanate films treated at 450 and 500 °C were crystallized forming pseudobrookite and landauite phase as shown by XRD patterns. The presence of urea in the synthesis procedure promoted the crystallization rate. XPS data indicated that iron ions existed in divalent and trivalent forms, and their ratio was changed for nitrogen-doped sample. The formation of the complexes between iron and urea during sol aging with the following reduction of Fe^3+^ to Fe^2+^ within calcination can be a reason not only for accumulation of iron onto the surface as shown by EDS but also for twice increase of divalent iron as registered by XPS. The iron titanate films extended the visible light absorption. Two band gap energy values for all iron-contained films were calculated. The photocatalytic response of all iron titanate films treated at 450 °C compared to pure titania films was spectacularly enhanced under UV and visible light. The slight enhance in photocatalytic activity of nitrogen-doped iron titanate films can be explained by the interstitial nitrogen incorporation rather than substitutional.

## Background

Development of photocatalysts with visible light sensitivity is an actual research at present due to the limited application of titania to outdoor application. Titania modification with a metal ion, such as Cr^3+^, Fe^3+^, V^5+^, Pt^4+^, or Ru^3+^/^4+^ [1–5] is one of the ways to overcome this problem as a result of narrowing the band gap or forming the metal sublevels within its band gap. However, cation doping often leads to the formation of defects, and improvement of the photocatalytic activity is limited. Attempts to improve the performance of metal ions contained TiO_2_ as a photocatalyst and to extend its light absorption are concerned with the effect of co-dopants. Iron is the most appropriated dopant agent as it has the radium identical to Ti^4+^ and the half-filled d electronic configuration.

There are numerous reports about the photocatalytic properties of iron-doped titania where the general roles are signed: there is an optimal concentration of iron into titania leading to the increase in photocatalytic rate; the higher the iron doping level, the lower the photoresponse due to the high recombination rate [6–8]. Only some papers connected with the synthesis and photocatalytic application of materials based on mixed oxides of titanium and iron with their high ratio forming iron titanates are presented. Among them, non-enzymatic fixation of molecular dinitrogen to ammonia under UV light, that is one of the important photosynthesis reaction, using the films composed on anatase and iron titanates demonstrating the perspective way to the application of such materials in ecological photocatalysis [1, 9, 10] Iron titanate-based nanocomposites is investigated at different pH, and the conditions used for the colloidal synthesis of the reactants. The formation of iron titanate phases on the grain boundaries of the binary oxide particles and the dependence of aggregates morphology on pH are demonstrated. It is shown that the relationship of the structure and properties of the materials, as well as the dependence on the preparation conditions and enhanced photocatalytic activity for the decomposition of organic dye. The materials demonstrated the high photocatalytic activity comparing with iron titanates produced by conventional techniques with strong magnetic separation properties [11]. As known from literature, the synthesis of iron titanates, Fe_2_Ti_2_O_7_, Fe_2_TiO_5_, FeTiO_3_, or others, can be achieved by ceramic or sol-gel technique [9–12]. One obvious finding is that the yield of the necessary product in a material is still low, and the iron titanates formation accompanied by metal oxides.

Nitrogen and iron ions co-doped titania was synthesized by sol-gel method using dodecylamine as both a structure-directing agent and a nitrogen dopant. Fourfold and double higher photocatalytic activity compared to P25 and N-doped titania, respectively, was reported for nitrogen co-doped with 1% iron in titania. The increase of iron content to 2% in the samples led to the decrease in the photocatalytic activity [13]. The synthesis of nitrogen- and iron-doped titania by hydrazine method is reported where the increase in NO photodestruction capability under both UV and visible light was noted [14].

Here, we propose the simplest and the fastest route of the synthesis of the mixture of iron titanate phases in the form of the films crystallized at a relatively low temperature and their optical, structural, and photocatalytic investigation. It is firstly reported that the application of urea in the synthesis of the films with high ratio of iron and titanium clarified the influence of the doping agent on the structural features.

It must be noted that the efficiency of a photocatalyst used in the environmental purification is determined not only by the quantum yield and rate of photodestruction of the pollutants but also by the versatile capability to effectively absorb both UV and visible irradiation as well as the need for a post-treatment removal of the catalyst. For this purpose, the photocatalysts in the form of thin films are most promising and convenient materials to be studied in this branch of ecological purification.

## Methods

Non-porous bare TiO_2_ and nitrogen-doped titania films were obtained from a sol contained titanium isopropoxide (Merck), perchloric acid (Aldrich), and absolute ethanol (SC Chimreactiv SRL) cooled down using the ice-bath. Urea was used as a source of nitrogen and was added to the sol in the concentration of 5 mol%.

Non-porous iron titanate films (Fe_x_Ti_y_O_z_) were synthesized according to procedure describe in [9]. An alcoholic solution of iron chloride (Aldrich) and titanium isopropoxide (Fe:Ti = 1:1) was heated at 40 °C with the continuous stirring, the obtained films were subjected to hydrolysis in humid air for 24 h before the calcination.

**Scheme 1:**
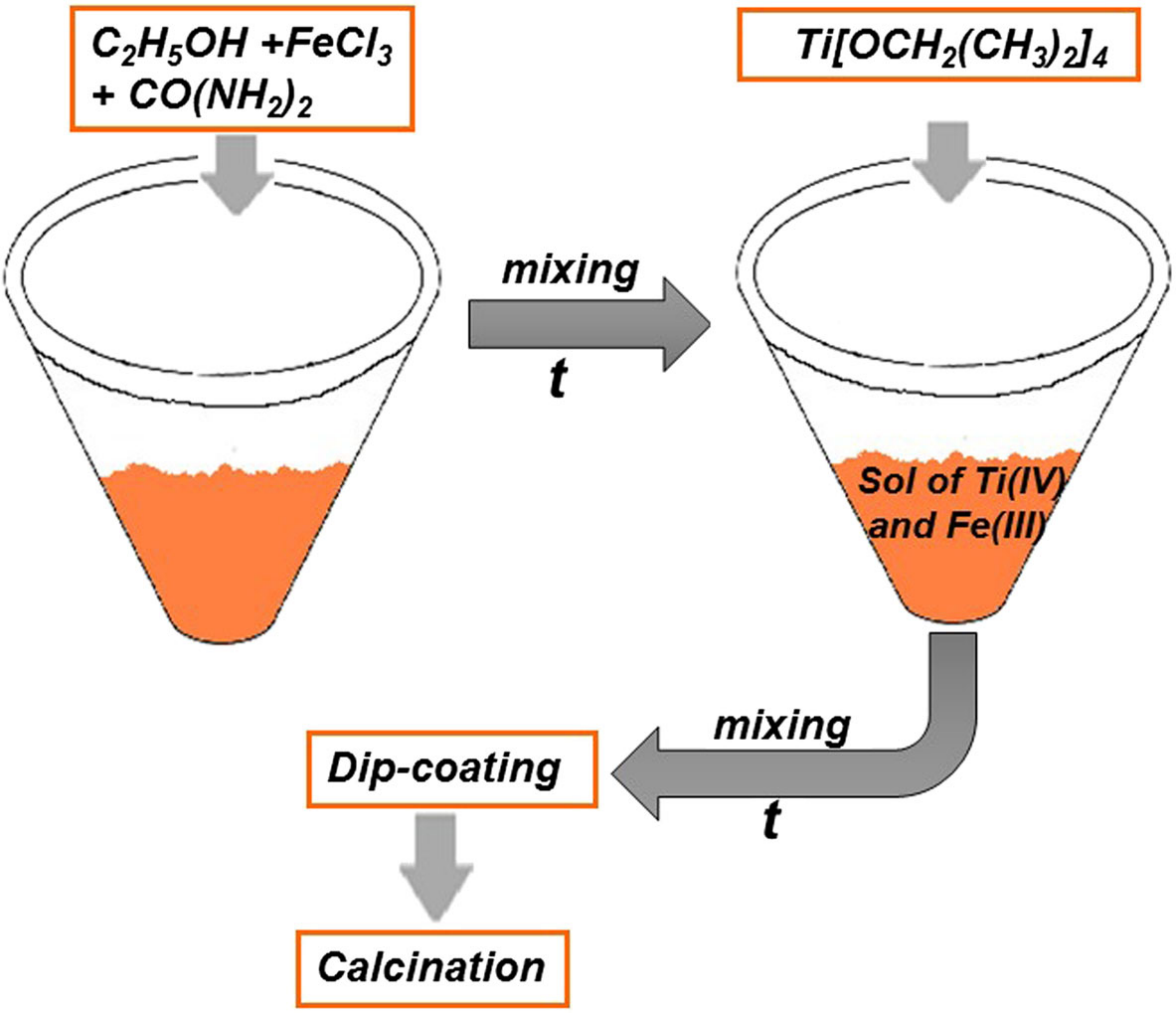
The illustration of the synthesis procedure of non-porous nitrogen-doped iron titanate films

Non-porous nitrogen-doped iron titanate films (N/Fe_x_Ti_y_O_z_) were obtained similarly to undoped films (Scheme 1): an appropriate amount of anhydrous iron chloride was dissolved in 25 ml of absolute ethanol previously heated to 40 °C and was stirred for 10 min till complete dissolution. The calculated amount of solid urea (5 mol%) and some drops of concentrated perchloric acid were added to the previously formed mixture. After mixing for 10 min, the calculated amount of titanium isopropoxide was added slowly. The molar ratio of elements was N:Fe:Ti as 0.05:1:1. The sol was ready for dip-coating after stirring for 15 min. The cleaned hot substrates were immediately used for dip-coating.

It must be noted that all the films were (1) dip-coated with the withdrawal speed of 1.5 mm/s; (2) coated on a glass substrate by three times: the first and the second layers were treated at 300 °C for 20 min, and the third layer was left for 24 h in humid air for hydrolysis; (3) the third layer was calcined (heating rate was 7 °C/min) at 450 or 500 °C for 20 min; and (4) the average mass of three-layered film was 0.006 g.

The applied substrates were purified by hydrofluoric acid for 10 s (silicon and titanium sheets) and by a soap (the glass slides) with the following prolonged washing with distillate water.

Photocatalytic activity evaluation of Fe_x_Ti_y_O_z_ and N/Fe_x_Ti_y_O_z_ films under UV and visible irradiation were estimated on the basis of two reactions: (i) reduction of toxic dichromate ions where 40 ml of aqueous solution contained potassium dichromate and disodium salt of ethylenediaminetetraacetic acid (Na_2_EDTA) in the molar ratio 1:1 adjusted to pH ≥ 2 by perchloric acid and (ii) degradation of tetracycline hydrochloride (TC) using 40 ml of an aqueous solution of 2 × 10^−5^ mol/L. The reaction temperature was kept constant (20 and 25 °C for i and ii reactions, respectively) during the experimental procedure. The change in Cr (VI) ions or TC concentration was monitored with a Lambda 35 UV-vis spectrophotometer (PerkinElmer) every 20 min; the conversion percentage was calculated from the change in absorption intensity at λ = 350 nm for Cr (VI) ions and λ = 357 nm for TC molecules. Prior to photocatalytic reactions, the adsorption equilibrium of film-liquid system was reached by stirring in the dark. A system was irradiated with a 1000 W middle-pressure mercury lamp for 120 min (dichromate reduction) and 90 min (TC degradation). The distance lamp-reactor was set at 90 cm. Two blank experiments were carried out: the catalytic reaction (dark condition),and the photoreaction (a bare glass was used instead of film). The photoreaction blank conversion percentage was extracted from the data obtained during photocatalysis. No significant changes in the absorption spectra of the liquid were observed for both blanks. For testing the visible light sensitivity, a filter transmitting light with λ > 380 nm was introduced in the photocatalytic setup.

Optical spectra were recorded with a double beam spectrophotometer (Lambda 35, PerkinElmer) within the wavelength range of 190–1200 nm.

The composites obtained on a silicon wafer were studied using a FEI Quanta Inspect S Scanning Electron Microscope, FEI Inspect S to get SEM images. Energy dispersive X-rays analysis (EDS) were performed using an instrument endowed with a liquid nitrogen-cooled SiLi detector from EDAX Co. mounted inside a FEI Quanta Inspect S Scanning Electron Microscope working under up to 30 kV acceleration voltage. For general estimations the EDS errors are at 1% for heavy elements and 2–3% for light elements (Be-F).

XRD patterns were performed on DRON-4-07 using Cu Кα irradiation (λ = 1,5418 Å). The peaks at 2*Ɵ* = 25.3° (undoped or doped by urea titania) and 2*Ɵ* = 29.6° (iron titanate films) were used to estimate a crystal size through application of the Scherrer equation. For XRD measurements, the film was scratched off.

The X-ray photoelectron spectroscopy (XPS) measurements were performed using a XPS spectrometer JSPM 4610 (JEOL) with MgKα (1253.6 eV) radiation source. The vacuum in the working chamber was 1 × 10^−7^ Pa. Width of the components, and the ratio contribution of Gaussian-Lorentzian distribution for certain atoms of the tested samples in the process of spectrum deconvolution were fixed. The component area was determined after background subtraction by Shirley method. Binding energies for the films were normalized with respect to the position of the C1s peak. The XPS binding energies were measured with a precision of 0.1 eV.

## Results and discussion

The activity of iron titanate films was estimated using two photocatalytic processes of the anthropogenic pollutant removal: (i) the tetracycline hydrochloride (TC) originated from extensive development of pharmaceutical industry, and the wide human consumption, its accumulation in the soil and water sources leads to the change in ecological equilibrium of our Earth and (ii) the dichromate ions derived from the chrome-plating and electronic industries. These processes include two different mechanisms where a photogenerated hole is involved in the TC degradation and a photoformed electron participates in the dichromate reduction. Comparing the conversion efficiency of both processes, the estimation of the photocatalytic properties of the films can be done. Based on the obtained data, it was concluded that the photocatalytic activity of the films is connected with the adsorption capability of the films that is, in turn, dependent on the annealing temperature.

**Fig. 1:**
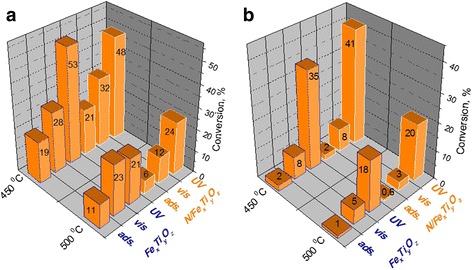
Adsorption level and photocatalytic activity of iron titanate films calcined at different temperatures in the processes of TC degradation (**a**) and dichromate reduction (**b**)

As seen from Fig. 1, the films treated at 450 °C were able to adsorb more TC molecules as well as dichromate ions. It must be noted that the conversion of TC during destruction was 18 and 6% as well as dichromate reduction was 10 and 3% under UV and visible light, respectively, over pure titania films. The photocatalytic response of undoped and nitrogen-doped iron titanate films treated at 450 °C compared to pure titania films is raised in more than 2.5 and 4 times under UV and visible light, respectively, in the case of TC destruction; more than 3.5 and 2.5 times under UV and visible light, respectively, during dichromate reduction. Moreover, photocatalytic performance under both UV and visible light is lowered almost in two times for the films treated at 500 °C contrary to 450 °C. It points that the change in surface and structural properties occur when the treatment temperature increased by 50 °C. It must be noted that the adsorption level and conversion percentage become lower for the undoped [15] and modified by urea iron titanate films treated at 600 °C. Comparing the activity of iron titanate films, its increase is noted for N/Fe_x_Ti_y_O_z_ treated at 450 °C in TC degradation process under visible light and dichromate reduction under UV light. For other cases, the activity was almost the same.

**Fig. 2:**
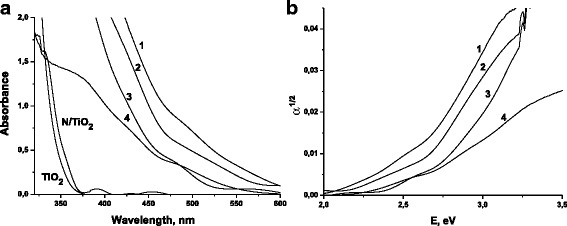
Absorption spectra (**a**) and the plot of square root of absorption coefficient vs incident photon energy (**b**) *1*, Fe_x_Ti_y_O_z_450; *2*, N/Fe_x_Ti_y_O_z_450; *3*, Fe_x_Ti_y_O_z_500; *4*, N/Fe_x_Ti_y_O_z_500

Optical absorption spectra of the films and the dependence of α^1/2^ vs E are reported in Fig. 2. The introduction of urea into titania leads to an insignificant shift of the absorption edge toward the long-wavelength region. All the iron titanate samples have an intense absorption band in the visible part of the spectrum comparing with TiO_2_ and N/TiO_2_ films. The shift towards higher wavelengths is in the order of N/Fe_x_Ti_y_O_z_500 < Fe_x_Ti_y_O_z_500 < N/Fe_x_Ti_y_O_z_450 < Fe_x_Ti_y_O_z_450.

All the iron titanate films exhibited the shoulder at a wavelength of around 460–580 nm (Fig. 2a) that can be attributed to the formation of the second phase as proposed in [16]. The band gap energy values were calculated assuming the indirect electronic transition through the relation between square root of absorption coefficient α and incident photon energy E (Fig. 2b). Comparing these values (Table 1), it is clear that the iron-contained films composed of two iron titanate phases; a separated titania phase is not formed. Additionally, the *E*_g_ values of N/Fe_x_Ti_y_O_z_ films become to be less comparing with Fe_x_Ti_y_O_z_ assuming the nitrogen incorporation.

**Table 1:** The band gap (*E*_g_,) values of undoped and doped with urea TiO_2_ and iron titanate films

Film	TiO_2_	N/TiO2	Fe_x_Ti_y_O_z_		N/Fe_x_Ti_y_O_z_	
T, °C	450	450	450	500	450	500
*E*_g_, eV	3.5	3.4	2.2; 2.5	2.3; 2.7	2.1; 2.5	2.2; 2.5

**Fig. 3:**
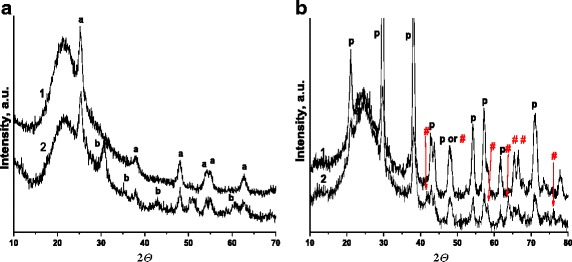
XRD spectra of the films (450 °C): **a** TiO_2_ (*a*,*1*), N/TiO_2_ (*a*, *2*), **b** Fe_x_Ti_y_O_z_ (*b*, *1*); N/Fe_x_Ti_y_O_z_ (*b*, *2*)

The structural essentials of bare titania and iron titanate as well as nitrogen-doped films (450 °C) are shown in the Fig. 3. Pure titania films contain anatase phase characterized by the diffraction peaks at about 25,4 (101), 38,9 (004), 48,1 (200), 54,1 (105), 54,9 (211), 62,4 (204), and 69 (213) (JCPDS 21-1272). Nitrogen-doped titania is mainly composed of anatase and brookite mixture yielding the additional reflections at about 30.8°, 35.2°, 42.8°, and 51.1° (Fig. 3a). The study of XRD graphs of bare and nitrogen-doped titanium dioxide shows that if the synthesis is carried out in the presence of urea then there is the acceleration of crystallization rate and phase transformation of anatase into brookite occurs while the average anatase crystallite size remains unchanged (about 14 nm). In the case of iron titanate films (450 °C), reflections from the anatase, rutile, or hematite phases in the XRD patterns are not observed while the diffraction peaks at about 21.1, 29.7, 38.2, 42.7, 54.3, 57.2, and 70.9 are indexed to pseudobrookite Fe_2_TiO_5_ phase. The appearance of 2*Ɵ* peaks at about 43.8, 47.8, 65.4, and 66.5 are signed to landauite phase (Fe_2_Ti_2_O_7_). Moreover, some additional diffraction peaks at about 41.6, 58.3, 63.9, and 75.9 corresponded to Fe_2_Ti_2_O_7_ phase [17] are fixed for N/Fe_x_Ti_y_O_z_ composite (Fig. 3b).

It was also reported [9] that pseudobrookite, anatase, and Fe_2_Ti_2_O_7_ phases were formed in the structure of one layered iron titanate films after heating at 600 °C. Recently, we have shown [15] the influence of the calcination temperature (450, 500, and 600 °C) of iron titanate films obtained in the same manner as the present films on their structural phase transformation. The XRD patters and the calculated *d* values clearly pointed on the transformation of pseudobrookite to landauite as well as the crystallization degree increase by reaching the calcination to 600 °C. The additional peak(s) was observed at 2*Ɵ* near 31.5 for Fe_x_Ti_y_O_z_ (500 °C); near 28.2, 32.0, 38.7, 41.8, 58.2, 63.9, 66.5, 74.2, and 75.9 related to Fe_2_Ti_2_O_7_ phase [15], and only some peaks belong to pseudobrookite were noted in the case of the iron titanate films treated at 600 °C. Hence, the higher fraction of Fe_2_Ti_2_O_7_ phase can be formed using urea pointing on the acceleration of phase transformation taking into account the brookite formation in the structure of urea-doped titania. The absence of titania crystalline phase (anatase or rutile) in XRD spectra (Fig. 3b) can be explained by gradual crystallization of every layer at 300 °C leading to the formation of crystallization centers that can cause to the preferential and faster crystallization of iron titanates rather than titania along. The crystallization degree of the films is varied in the range of N/Fe_x_Ti_y_O_z_ (450 °C) < Fe_x_Ti_y_O_z_ (450 °C) < Fe_x_Ti_y_O_z_ (500 °C), though the average crystal size estimated from the highest peak located at 2*Ɵ* = 29° by the Scherrer equation and belonged to pseudobrookite is ca.16 nm for all iron titanate composites.

In order to understand in depth this phenomenon, we further examined the effect of the synthesis conditions on the phase formation. Gupta et al. reported on [17] the study of ilmenite oxidation in argon and oxygen in the range of 700–1000 °C. It is concluded that the ilmenite is oxidized to Fe_2_Ti_2_O_7_. The further its transformation occurred to pseudobrookite and titania afterwards the Fe_2_TiO_5_ decomposition to Fe_2_O_3_ and TiO_2_ took place at temperatures lower than 800 °C. The similar results were published in [18] where the thermal oxidation of commercial ilmenite at 500–950 °C was reported. After heating at 500 °C, the main product of the reaction was pseudorutile (Fe_2_Ti_3_O_9_), although no complete oxidation of ilmenite occurred. By increasing the temperature up to 700 °C, the intensity of ilmenite and pseudorutile peaks decreased while the new phase of Fe_2_Ti_2_O_7_ appeared. X-ray diffraction patterns approved the presence of pseudobrookite, rutile, hematite, and Fe_2_Ti_2_O_7_ phases at 950 °C. The interaction of binary oxide nanoparticles, Fe_2_O_3_,and TiO_2_ in the ratio of 1:1 or 2:1 using the different synthesis pathways employed to obtained Fe_2_Ti_2_O_7_ or Fe_2_TiO_5_, respectively, in order to investigate the photocatalytic activity of heterostructures was reported [11]. The crystallization of pseudobrookite, landauite, anatase, and even hematite took place over reported conditions. It is argued that the crystallization of Fe_2_Ti_2_O_7_ or Fe_2_TiO_5_ occurred for all non-annealed samples as shown by XRD spectra. The crystallization degree is increased with the treatment temperature to 700 °C. Thus, the synthetic route proposed by us allow us to obtain the materials composed with the desired iron titanate phases without pure titania that can be achieved by the simple change of the synthesis conditions and/or calcination temperature.

**Fig. 4:**
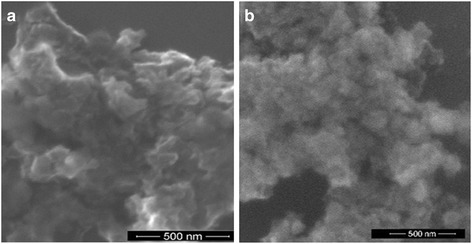
SEM images of Fe_x_Ti_y_O_z_ (**a**) and N/Fe_x_Ti_y_O_z_ (**b**) films treated at 450 °C

**Fig. 5:**
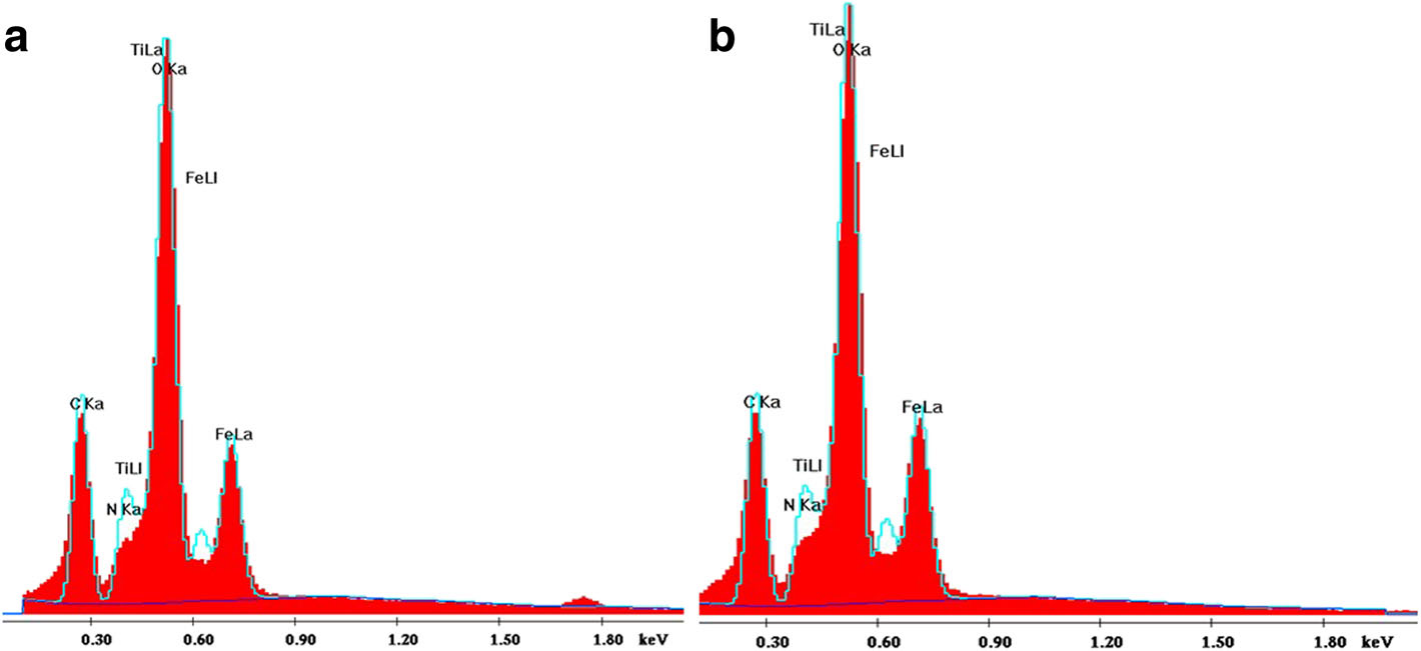
EDS images of Fe_x_Ti_y_O_z_ (**a**) and N/Fe_x_Ti_y_O_z_ (**b**) films treated at 450 °C

SEM images of the samples are shown in Fig. 4. The micrographs reveal that nanosized primary particles with spherical shape are formed. The EDS area scanning technique (Fig. 5) was used to detect the ratio of the elements on surface of the bare and nitrogen-doped iron titanate composites heated at 450 and 500 °C (Table 2). It can be seen that the ratio of metals onto the surface correlates with desire 1 to 1 in Fe_x_Ti_y_O_z_450 sample while iron atomic percent decreases for the film obtained at 500 °C. It must be noted that the surface was enriched by 10% of iron in the case of N/Fe_x_Ti_y_O_z_ for both temperatures (Table 2).

**Table 2:** The atomic percentage and ratio of the elements of the films obtained by EDS technique

Element	Fe	Ti	O	Fe:Ti:O
Film	At,%			
N/Fe_x_Ti_y_O_z_ 450 °C	20.63	19.19	38.74	1.1:1.0:2.0
Fe_x_Ti_y_O_z_ 450 °C	18.99	19.43	39.3	1.0:1.0:2.0
N/Fe_x_Ti_y_O_z_ 500 °C	21.61	20.95	42.23	1.0:1.0:2.0
Fe_x_Ti_y_O_z_ 500 °C	19.64	20.91	40.86	0.9:1.0:2.0

It was found [6] that the surface doping is dominant for low-level doping, and more Fe ions will enter TiO_2_ bulk with the increase of doping concentration. Contrarily, the high level doping proposed by us leads to the saturation of the surface by iron ions. Unexpectedly, the low oxygen ratio to metals was detected by EDS suggesting the formation of non-stoichiometric structure onto the film surface.

**Fig. 6:**
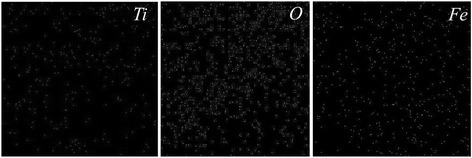
Kα X-ray mapping of Fe, O, and Ti over the surface of N/Fe_x_Ti_y_O_z_ film treated at 450 °C

X-ray Kα mappings of iron, titanium, and oxygen are shown in Fig. 6. The uniform distribution of main elements is observed. These three maps confirm the forgoing EDS analyses.

XPS survey spectra recorded on a wide binding energy range (0–1200 eV) were used to determine the chemical state of the elements. Ti2p peaks contained Ti2p_3/2_ and Ti2p_1/2_ subpeaks due to the spin orbital coupling effect located at 459.2 and 464.8, respectively (Table 3). The binding energy difference of 5.6 eV in both Fe_x_Ti_y_O_z_ and N/Fe_x_Ti_y_O_z_ films revealed a valence state +4 for Ti, indicating the presence of titanium atoms in the form Ti^4+^. It is known that the XPS lines belong to Ti2p_3/2_ component at 458.2–458.9 eV are attributed to the Ti (IV) species of the pure titania crystalline network and signed to a titanium atom surrounded by two oxygen atoms [19]. We observed the shift of Ti2p components towards the higher binding energy that is explained by formation of the Fe–O–Ti bonds. The similar XPS Ti2p shift were reported in [20–23] where the higher iron content in titania, the higher BE value. Hence, the iron ions do have an influence on the position of Ti2p peaks, indicating the change of coordination and structure of lattice Ti^4+^ ions.

**Table 3:** Binding energies of the elements detected by XPS and their relative intensity values

Film (450 °C)	N/Fe_x_Ti_y_O_z_		Fe_x_Ti_y_O_z_	
XPS peak	BE, eV	Relative intensity, %	BE, eV	Relative intensity, %
Fe2p_3/2_	707.7	43.1	707.6	22.3
	710.5	56.9	710.3	77.7
N1s	397.2	63.3	–	–
	398.3	28.0		
	399.8	8.7		
Ti2p_3/2_	459.3	100	459.2	100
O1s	531.0	94.3	531.1	96.5
	532.5	5.7	532.9	3.5

**Fig. 7:**
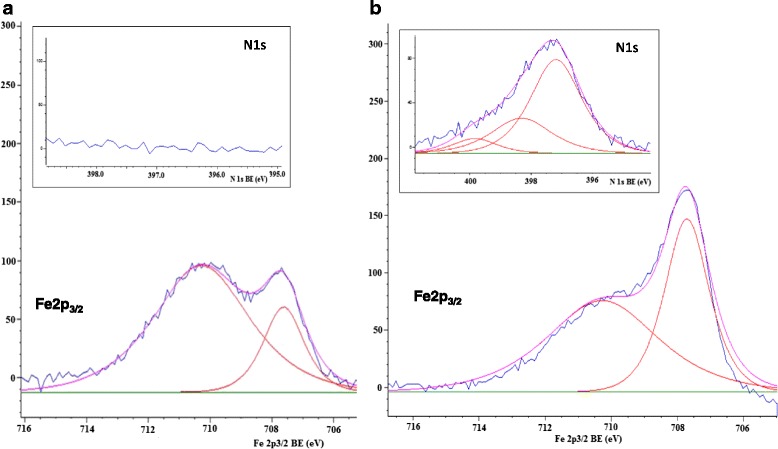
Deconvoluted XPS Fe2p_3/2_ spectra of Fe_x_Ti_y_O_z_450 (**a**) and N/Fe_x_Ti_y_O_z_450 (**b**) films. *Inset*: XPS N1s region

The Fe2p_3/2_ and N1s core spectra of undoped and doped with urea Fe_x_Ti_y_O_z_ films are presented in Fig. 7. An asymmetric Fe2p_3/2_ line with two deconvoluted maxima at 707.7 and 710.3 eV is fixed for both composited. The peak located at the lower BE is attributed to Fe^2+^, and the second component to Fe^3+^ ions. In the case of Fe2p region, the lower binding energy values are explained by the formation of combined bonds with titanium atoms [21]. Nevertheless, only about 22% of Fe^2+^ is detected on the surface of Fe_x_Ti_y_O_z_450 whilst twice higher divalent iron content is noted for N/Fe_x_Ti_y_O_z_ sample. The appearance of some peaks attributed to divalent and trivalent iron in 1%Fe_2_O_3_/P25 was also detected [24] using the FeCl_3_, Degussa P25 and ammonia during doping procedure where the peak located at 709.0 eV was attributed to Fe^2+^ in FeO or Fe_3_O_4_. The deconvoluted XPS peak at 707.75 eV attributed to Fe^2+^ ions was also registered for the iron-doped titania nanopowders obtained by sensitized IR laser pyrolysis [25]. Thus, we suggest that the crystallization of Fe_2_TiO_5_ and Fe_2_Ti_2_O_7_ phases concurrently with the reduction of iron (III) to iron (II) ions by the formed carbon-, chlorine- and even nitrogen-contained species, in the case of urea-doped films, occurred during thermal treatment [25, 26].

The narrow O1s peak registered for both samples was deconvoluted to the peaks at about 531 and 533 eV corresponded to the lattice oxygen in the metal oxides and adsorbed water [21]. In general, the shift of Ti2p_3/2_ and O1s XPS maxima to higher binding energy caused by a subsequent change in the charge distribution of the atoms due to the incorporation of iron atoms proves the formation of Fe–O–Ti bonds [6, 12, 22].

The nature of the nitrogen species formed in nitrogen-modified titania is still under debate. XPS values within 396–397 eV are signed to the formation of the bond corresponded to N–Ti–N bonds [27, 28] and O-Ti-N [28, 29], respectively. N1s binding energies of 399.1 and 400.5 eV measured for modified by urea commercially available TiO_2_ powder were assigned to carbon nitrides (399–400 eV, C = N-C), graphite-like phases (400.6 eV, N-Csp^2^) and to polycyanogen (399.0, 400.5 eV, (−C = N−)_x_) [30].

XPS N1s peak with three deconvoluted maxima was fixed for N/Fe_x_Ti_y_O_z_450. Binding energy positions at 397.2, 398.1, and 399.8 eV provide the assumption about an interstitial nitrogen incorporation with the trace of remained nitrogen-carbon bonds. Analyzing all our previous investigations connected with the nitrogen and metal co-doped titania films, it is concluded that interstitial nitrogen incorporation occurred when urea was applied as the doping agent [31, 32], On the other hand, the substitutional nitrogen incorporation performed by Pulse laser deposition method is responsible for the improvement of photocatalytic performance for nitrogen and zirconia co-doped titania films [28].

## Conclusions

The synthesized undoped and nitrogen-doped iron titanate films with the high ratio of iron to titanium were tested as the potential photocatalysts to remove organic and inorganic contaminations. The photocatalytic response of all iron titanate films treated at 450 °C compared to pure titania films is sharply enhanced for both reactions under UV and visible light. The slight enhance in photocatalytic activity of nitrogen-doped iron titanate films compared to undoped iron titanate can be explained by the interstitial nitrogen incorporation. Nevertheless, the structural properties of the films are certainly changed in the presence of this doping agent, and the films contained pseudobrookite as well as landauite can be used for the application in ferroalloy industry and catalytic reaction, for instance chemical looping combustion and methane decomposition. It is suggested that urea addition to the iron titanium contained sol led to the shift of the reaction equilibrium towards Fe_2_TiO_5_ and Fe_2_Ti_2_O_7_ formation rather than to oxygen substitution by nitrogen. The appearance of the peak attributed to divalent iron was detected for Fe_x_Ti_y_O_z_ films as shown by XPS. Twice increase in the relative intensity of Fe^2+^ in N/Fe_x_Ti_y_O_z_ surface can be interpreted by the formation of the complex compound between urea and iron leading to the saturation of the surface by iron ions (proven by EDS), and the appropriate conditions for iron reduction. It should be stressed that the higher content of divalent iron could be related to the appearance of more XRD peaks belong to landauite phase. The iron titanate films showed high absorption in the visible part of the spectrum and, consequently, the band gap energy values are decreased. The calculated two band gap energy values for all iron contained films are an additional evident of two phase formation. The synthesis procedure proposed herein for iron titanate films can be more potential for practical application in comparison with the traditional sol-gel method due to the fast and the lower cost of synthesis process.

## Abbreviations

EDS: Energy dispersive X-ray spectroscopy
Fe_x_Ti_y_O_z_: Iron titanate film
N/Fe_x_Ti_y_O_z_: Nitrogen-doped iron titanate film
Na_2_EDTA: Sodium ethylenediaminetetraacetate
SEM: Scanning electron microscopy
TC: Tetracycline hydrochloride
UV: Ultra-violet
XPS: X-ray photoelectron spectroscopy
XRD: X-ray diffraction
